# Isolation, morphological, and molecular characterization of a native *Heterorhabditis indica* strain from the Mid-Indian Himalayas with insights into biocontrol potential

**DOI:** 10.3389/fpls.2025.1576159

**Published:** 2025-06-19

**Authors:** Ashish Kumar Singh, Jagadeesh Patil, Aarthi Nekkanti, Amit U. Paschapur, Sunaullah Bhat, Vinita Gouri, Gaurav Verma, Mukesh Jaiman, Pankaj, K. K. Mishra, Lakshmi Kant

**Affiliations:** ^1^ Crop Protection Division, Indian Council of Agricultural Research (ICAR)-Vivekanada Parvatiya Krishi Anusandhan Sansthan, Almora, Uttarakhand, India; ^2^ Division of Nematology, Indian Council of Agricultural Research (ICAR)-Indian Agricultural Research Institute, New Delhi, India; ^3^ Division of Germplasm Collection and Characterisation, Indian Council of Agricultural Research (ICAR)-National Bureau of Agricultural Insect Resources, Bengaluru, Karnataka, India

**Keywords:** entomopathogenic nematodes (EPNs), biocontrol agents, *Spodoptera frugiperda*, *Anomala dimidiate*, *Spodoptera litura*, *Spilosoma obliqua*

## Abstract

Entomopathogenic nematodes (EPNs) from the families Steinernematidae and Heterorhabditidae are biocontrol agents for the management of a wide range of insect pests. There is a tremendous opportunity for the discovery of new nematode strains and species adapted to local environmental conditions and insect pests. Therefore, in the present study, efforts were made to isolate EPN strains/species from the Northwestern Himalayas (NWH) region. The soil samples were collected from different locations in Almora, Uttarakhand. The collected soil samples were baited with *Corcyra cephalonica*, and they were observed regularly for their mortality. EPNs were isolated from the cadaver using the white trap method. Based on morphological and morphometrical studies, *Heterorhabditis* sp. *VLEPN01* shows a resemblance to the species of *Heterorhabditis indica*. Further identity was confirmed with molecular characterization using the rDNA ITS marker. The sequence of this native EPN isolate revealed 99.87% similarity with *H. indica* isolated from Mizoram, Northeastern India (MF618314). The efficacy of EPN was evaluated against major insect pests of the NWH region. The results showed that *H. indica VLEPN01* is capable of causing 100% mortality in fall armyworm (*Spodoptera frugiperda*), white grub (*Anomala dimidiata*), tobacco caterpillar (*Spodoptera litura*), and pod borer (*Helicoverpa armigera*) under laboratory conditions. Hence, *H. indica VLEPN01* can be utilized for field testing in the management of insect pests occurring in the NWH region.

## Graphical Abstract

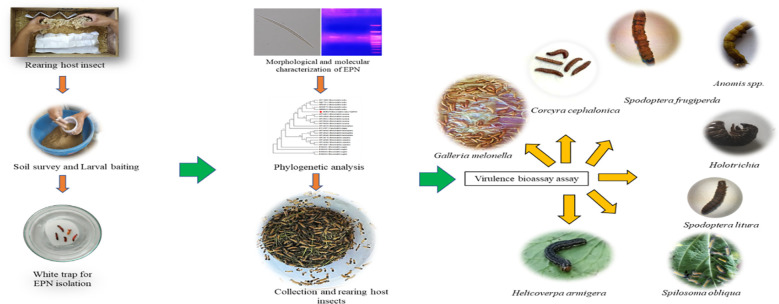

## Highlights

- A new strain of *Heterorhabditis indica* (*VLEPN01*) was isolated from the Mid-Indian Himalayas using *Corcyra cephalonica* baiting.
- The *VLEPN01* strain exhibited unique morphological and morphometric traits compared to the Indian isolate (CH7) and type specimen, with significant differences in morphometry.
- The ITS-region-based sequence analysis (786 bp, accession ID OK001870) confirmed 99.87% similarity with *H. indica* from Mizoram, placing VLEPN01 in a distinct phylogenetic clade.
- The successful identification and characterization of *H. indica* VLEPN01 highlight its potential for use in sustainable pest management programs in the Himalayan agroecosystem.

## 1 Introduction

The Indian Himalayan region extends from the Northwestern Himalayan (NWH) states (Jammu and Kashmir, Ladakh, Himachal Pradesh, and Uttarakhand) to the northeastern states (Sikkim, Arunachal Pradesh, Meghalaya, Nagaland, Manipur, Mizoram, Tripura, and Assam). Land resources in Himalayan regions support the farming-based community for raising crops, animal husbandry, fruit plantation, agro-pastoral, and forest (Partap, 1999). Variations in the commercialization of agriculture and the cultivation of subsistence crops are turning into a major cause of migration and leading to the formation of ghost villages in the hill districts of Uttarakhand (Trivedi, 2012; Dewari, 2019). Thus, adopting new, emerging cropping-system-based technologies to cultivate high-value fruits, vegetables, millets, saffron, ornamentals, medicinal plants, and export-value crops are the key sectors in ensuring food, nutritional, and financial security (Barah, 2010).

Lepidopteran and coleopteran pests like white grub (*Holotrichia* spp.), fall armyworm (*Spodoptera frugiperda*), tobacco caterpillar (*Spodoptera litura*), pod borer (*Helicoverpa armigera*), cutworm (*Agrotis ipsilon*), and hairy caterpillar (*Spilosoma obliqua*) are polyphagous pests attacking a wide range of crops, including pulses, vegetables, and cereals (Thakur et al., 2012; Paschapur et al., 2023). The invasion of fall armyworm has been fierce lately and is an emerging pest to maize crops. Application of insecticides is one of the most common practices adopted by farmers in managing insect pests in various regions, including the NWH region (Sharma et al., 2022). However, the detrimental effects posed by the indiscriminate use of insecticides include insecticide resistance and negative impacts on non-target organisms and the environment (Kumari and John, 2019; Poudel et al., 2020). These concerns demand alternative management practices that are target-specific and highly efficient without compromising on environmental safety.

One such alternative management practice is the use of biological control agents, including both microbials and macrobials. Among the macroorganisms, entomopathogenic nematodes (EPNs) are one of the successful biological control agents in managing many insect pests of different orders. The EPNs from two families, Steinernematidae and Heterorhabditidae, are the potential biocontrol agents for the management of a wide range of insect pests (Dillman and Sternberg, 2012; Askary, 2022). They are mutually associated with gram-negative symbiotic bacteria, *Xenorhabdus* and *Photorhabdus*, respectively (Grewal and Georgis, 1999; Mohan et al., 2003). Infective juveniles of the EPNs freely move into the soil and enter into the host’s body through natural openings (mouth, anus, and spiracles) in the insect body. The bacteria multiply and release toxins that are harmful to insects, and the death of the insect is caused by hemocelic septicemia (Woodring and Kaya, 1988; Ahantarig et al., 2009). The EPN-infected insect dies generally within 24–48 h, and the application of EPNs does not impose any non-target impacts.

Despite the advantages of using EPNs, very few attempts have been made to isolate and characterize EPNs from the NWH region, and their biocontrol potential is also a relatively underutilized resource in insect pest management in the NWH region. Hence, the current research was conducted with the objectives to isolate indigenous EPNs from the Kumaon region of the NWH agro-ecosystem and to characterize them by morphology, morphometry, and molecular methods. Moreover, the biocontrol potential of isolated EPN against major insect pests of the NWH region—fall armyworm (*Spodoptera frugiperda*), white grub (*Anomala dimidiate*), tobacco caterpillar (*Spodoptera litura*), pod borer (*Helicoverpa armigera*), and looper *Anomis involute*—has been evaluated.

## 2 Methodology

### 2.1 Source of host insect

Larval-stage *Corcyra cephalonica* samples were obtained from infested wheat flour. The *C. cephalonica* samples were mass-reared on an artificial diet of broken corn seeds in a rectangular wooden box having a dimension of 43 cm × 29 cm × 14 cm (length/width/height) fitted with an iron mesh (Karunarathne et al., 2020). Adult *Galleria melonella* samples were collected from bee boxes and reared on an artificial diet (**Supplementary Table S1**) by inoculating the eggs at 25 ± 2°C with 14 h of photophase and 10 h of scotophase. Third-instar white grub larvae were collected from the infested soybean fields from Experimental Farm, ICAR-Vivekananda Parvatiya Krishi Anusandhan Sansthan, Hawalbagh, Almora (29.63° N and 79.63° E, 1,250 mamsl) by digging through with a shovel, and these were transported to the laboratory in sterilized 0.25-L aerated plastic containers (with potato slices). In the laboratory, the white grubs were surface-sterilized twice with 70% ethyl alcohol and twice with autoclaved double-distilled water to remove any surface contamination and reared at a temperature of 25 ± 2°C and relative humidity of 65 ± 5% for 48 h by releasing them in a large sterile cylindrical plastic container (60 × 75 cm) containing autoclaved farm yard manure and sandy loam soil at a ratio of 1:1 (Sushil et al., 2008). The grubs were regularly fed with surface-sterilized potato slices to maintain a robust white grub population.

Adult (male and female) *Spodoptera litura*, *Spilosoma obliqua*, *Helicoverpa armigera*, and *Anomis involuta* were collected directly from the infested field of Experimental Farm, ICAR-Vivekananda Parvatiya Krishi Anusandhan Sansthan, Hawalbagh, Almora (29.63° N and 79.63° E, 1,250 mamsl) by using a light trap and identified based on morphological features following Specht et al. (2013); Brambila (2013), and Lal et al. (1994). The collected adults were placed in a plastic container covered with muslin cloth and fed with a sucrose solution. The container was placed at 25 ± 1°C with 14 h of photophase and 10 h of scotophase. Laid eggs in the muslin cloth were collected and placed in another plastic container added with a natural food source (tomato leaves; *S. obliqua* were reared on a soybean host) for the hatched larvae.

### 2.2 Survey, soil sample collection, and baiting

Soil samples were collected from forest land, agricultural land, and orchards during the period June–October 2020 in Almora and Nainital districts, Uttarakhand, India (**Supplementary Table S2**). It was anticipated that the microbial diversity would flourish well post-monsoon season; therefore, sampling was performed in June–October. However, for the EPN-positive site, sample was collected in August. A total of 50 g of soil sample was collected using a hand shovel (*khurpi* and *kutla*) at a depth of 20–30 cm and pooled to get a composite sample of 200 g. The samples were pooled in a polyethylene bag (Himedia), labeled with information on the place of collection, type of crop, annual rainfall, and climatic condition, carried to the laboratory in a styrofoam box, and kept overnight at room temperature until processing. The geo-coordinates of the locality were mapped using the Android mobile application (exa mobile 2.2.05.249).

The samples were processed manually to remove gravels and made into fine granules. A portion of the soil sample was analyzed for physicochemical parameters. Nearly 50 g of the processed soil samples was placed in each plastic container (height, 10 cm; diameter, 5 cm), and five to six late-instar larvae of *Corcyra* were placed in these containers. The containers were placed inverted in a plastic tray, covered with white cloth, and incubated at 25°C for 8 days. Each container was examined for larval mortality by probing the larvae with a needle to observe their mortality and motility. The cadavers without odor and rotting as well as the red-colored body were considered as samples of EPN infection. These cadavers were collected and surface-sterilized using 80% ethanol, followed by washing with sterile distilled water. The cadaver was transferred to a white trap (White, 1927) for collection of emerging EPNs. The infective juveniles (IJs) that emerged were stored in Ringer’s solution at a density of 5,000 IJs/mL at 15°C (Gowda et al., 2020). The pathogenicity of EPNs to prove Koch’s postulate was tested using the late instar of *Corcyra* and *Galleria* larvae against the dosage of 500 IJs/mL to find a similar sign of mortality (Pelczar and Reid, 1972; Kaya and Stock, 1997).

### 2.3 Morphology and morphometry of native entomopathogenic nematodes

Isolated EPNs were inoculated on the late-instar larvae of *Corcyra cephalonica* at the rate of 100 IJs larva^-1^ and incubated at 25°C in a dark chamber to study the morphological and morphometric parameters. First- and second-generation adults were collected from 3- and 5-day-old cadavers by dissecting in Ringer’s solution (Mhatre et al., 2017). Samples of all stages were heat-killed by using the hot triethanolamine and formaldehyde fixative and Seinhorst solution I and II, following the methodology of Seinhorst (1959). The samples of different life stages were picked and mounted in dehydrated glycerine on a glass slide (76 × 25 mm) and deployed for detailed microscopic studies (Poinar, 1990). Morphometric measurements, morphological details, and imaging were performed in a trinocular research microscope (Carl Zeiss Microscopy GmbH provided with DIC optics). A total of 20 specimens of each life stage were examined for taxonomic studies. The obtained data for isolated specimens were correlated and compared with the original/earlier description of the specimens (Poinar et al., 1992).

### 2.4 Molecular characterization of native entomopathogenic nematodes

The genomic DNA was extracted from a single female of isolated entomopathogenic nematodes following a method described by Joyce et al. (1994). The ITS region of each isolated EPN was amplified by polymerase chain reaction (PCR) using a forward primer (5′-TTGATTACGTCCCTGCCCTTT-3′) and a reverse primer (5′-TTTCACTCGCCGTTACTAAGG-3′) (Vrain et al., 1992) containing 10 μL of the DNA, 0.5 μL dNTP mixture (10 mM each), 10× PCR buffer containing MgCl_2_, 0.3 μL Taq polymerase, 0.5 μL (100 pM/μL) of each primer, and 10.7 μL double-distilled water. The amplified products were separated and visualized under 1.5% agarose gel electrophoresis. The amplified PCR product was purified with the Qiagen Gel Purification Kit, and the same was sequenced by Sanger’s method (Biologia Research India Pvt. Ltd., Karnal, Haryana, India). The obtained sequences were aligned and edited using BioEdit with the sequences of related strains. Finally, the edited sequences were submitted to NCBI to get the accession number. Subsequently, the phylogenetic tree was constructed from ITS DNA sequences of native EPN species and related species retrieved from NCBI in MEGA 7.0 by the neighbor joining method (Kumar et al., 2016).

### 2.5 Principal component analysis of the morphometric data of different life stages

The dataset for this study comprises data from our isolate *VLEPN01* and additional data sourced from previous studies. To ensure comparability across variables, all data were standardized by converting them into z-scores. PCA was conducted using XLSTAT 2024.2.2.1422. We utilized the correlation matrix to account for differences in variable scales. The PCA was configured to extract up to a maximum of five factors, and the correlation biplot with automatic coefficient settings was used for visualization. Eigenvalues were calculated to assess the variance explained by each principal component, while eigenvectors were extracted to evaluate the influence of each variable on the principal components.

### 2.6 Evaluation of the biocontrol potential of native EPN species against insect pest

In the current study, the biocontrol potential of *H*. *indica VLEPN01* was evaluated against the third-instar larvae of *S. litura*, *S. obliqua*, *H. armigera*, *S*. *frugiperda*, and *Anomis involuta*. The experiment was conducted in a 9-cm Petri dish with a depth of 1.8 cm. Nearly 20 g of sterilized soil with 15% moisture content was added to each Petri dish. Subsequently, these Petri dishes were inoculated at different concentrations (10, 25, 100, 500, 1,000, and 2,500) of IJs. After 4 h of IJ inoculation, the third-instar larvae of each insect species were placed in a Petri dish, and fresh leaf discs were offered as food to each larva. Each experiment was repeated five times, and the recommended insecticide, emamectin benzoate (0.3 g/100 mL water), and sterile distilled water were used as positive and negative controls, respectively. The mortality of the larvae was recorded every 24 h, with death confirmed by the presence of EPN observed in the dissected cadavers under a stereomicroscope.

In the laboratory, the white grubs were surface-sterilized twice with 70% ethyl alcohol and twice with autoclaved double-distilled water to remove the surface contamination and reared at a temperature of 25 ± 2°C and relative humidity of 65 ± 5% for 48 h by releasing them in a large sterile cylindrical plastic container (60 × 75 cm) containing autoclaved FYM and sandy loam soil at a ratio of 1:1. The grubs were regularly fed with surface-sterilized potato slices to maintain a healthy and robust white grub population. For the bioassay, autoclaved FYM and sand-mixed soil were impregnated with multiple concentrations of IJs and placed in a 100-mL plastic container. Five larvae were released at different depths in each container. The observation of mortality was undertaken at 5 days post-treatment as per Bhatnagar et al. (2004). Then, 2 mL/L of chlorpyrifos was taken as a positive control.

### 2.9 Statistical analysis

The statistical analysis of the data was conducted using appropriate methods for each experiment. For PCA of morphometric data, XLSTAT 2024.2.2.1422 was utilized to perform the PCA, employing the correlation matrix to account for variable scale differences. Eigenvalues and eigenvectors were calculated to assess the variance explained by each principal component and to determine the influence of each variable on these components. The correlation biplot with automatic coefficient settings was used for the visualization of PCA results. For the virulence bioassays, the data were analyzed using analysis of variance (ANOVA) followed by *post-hoc* tests for multiple comparisons using the Web-Based Agricultural Statistics Software Package (Jangam and Thali, 2002). Mortality data were subjected to statistical analysis to compare the efficacy of different concentrations of EPNs and control treatments. Mean mortality rates were compared using Tukey’s HSD test to determine significant differences among treatments. In all cases, statistical significance was determined at a 5% level (*p* ≤ 0.05). Data were presented as means ± standard error (SE) as appropriate.

## 3 Results

### 3.1 Soil sample collection and baiting

In this study, a total of 40 soil samples were collected to investigate the presence of EPNs. The climate data at the location of the soil samples collected during the month of August 2020 were obtained from the Indian Meteorological Department. We recorded an average monthly rainfall of 351.07 mm (in the month of August), a mean maximum temperature of 29.71°C, and a mean minimum temperature of 22.39°C. Among these, one sample tested positive for EPN recovery, as confirmed by observing a dead, brick-red EPN-infected *C. cephalonica* larva at 7 days post-baiting. These positive samples were located at geo-coordinates 79°37′52.071″ E, 29°38′0.783″ N, at an elevation of 1,220 meters above sea level (MASL). The habitat was identified as an agro-ecosystem associated with fodder crops, and white grub insects were observed at a soil depth of 30–40 cm. The soil texture is sandy loam (pH 6.59, EC 0.105 dSm^-^¹, organic carbon 10.6 g kg^-^¹). The available nitrogen, phosphorus, and potassium contents were 467.6, 49.32, and 128.2 kg ha^-^¹, respectively.

### 3.2 Morphological characterization of *Heterorhabditis indica*

**Figure 1:**
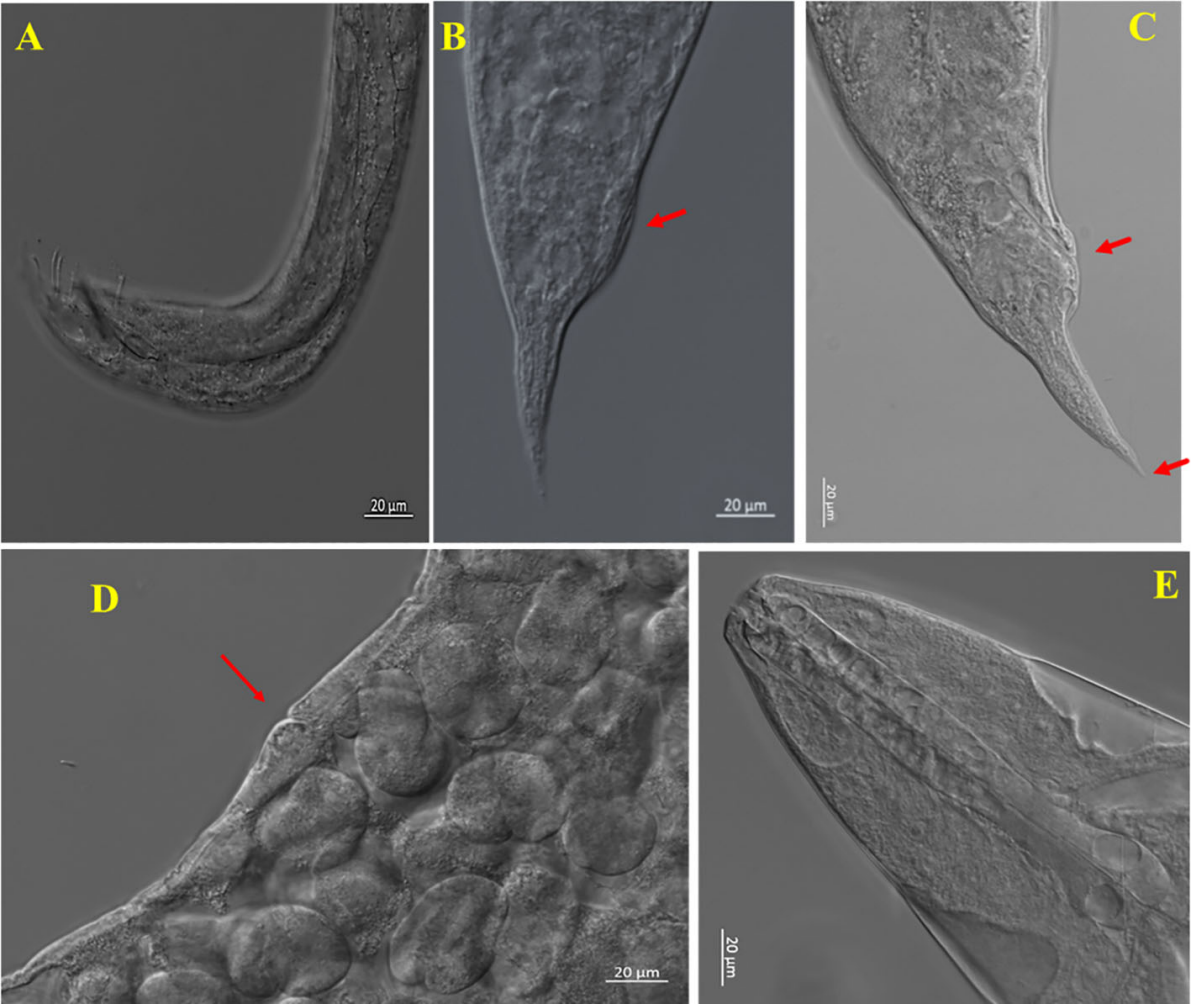
**(A)** Light microscopy photographs of male tails showing bursal papillae. **(B)** First-generation-female tail with the arrow showing anal swelling. **(C)** First-generation female with the arrow showing the excretory pore opening and tail end. Vulva of an hermaphrodite with the arrow showing it as slightly protruding **(D)**. Anterior end of an hermaphrodite **(E)**.

The morphological characterization of *Heterorhabditis indica* strain *VLEPN01* across various developmental stages was conducted using light microscopy, revealing distinct anatomical features that aid in species identification and differentiation (**Figure 1**). The light microscopy (LM) photographs of male *H. indica* tails highlight the prominent bursal papillae (**Figure 1A**). These papillae are well defined and evenly spaced along the tail, contributing to the male sample’s reproductive function. The arrangement and structure of these papillae are critical for accurate species identification. The tail region of a first-generation female *H. indica* exhibited notable anal swelling, a key feature indicating reproductive maturity. Additionally, the tail end of the female sample showed the location of the excretory pore opening. This feature is essential for species identification and provides insight into the nematode’s physiological characteristics. The vulva of the hermaphrodite was slightly protruding, distinguishing it from other developmental stages. This protrusion is significant for understanding the reproductive anatomy of the hermaphrodite. The anterior end of the hermaphrodite, including the head and esophageal structures, was also visible. These features are crucial for comprehensive morphological characterization. The morphological analysis provided a detailed view of *H. indica VLEPN01* across different developmental stages. The images illustrate key anatomical structures, including the bursal papillae in male samples, anal swelling and excretory pore location in female samples, and the reproductive and anterior structures in hermaphrodites. These observations are integral for the accurate identification and understanding of the species’ biology.

### 3.3 Morphological and morphometrical characterization of *Heterorhabditis* strain *VLEPN01* at various developmental stages

In the morphological study of *H. indica*, the first-generation female sample exhibited a tail with distinct anal swelling, as shown in **Figure 1B**. This feature is characteristic of the species and aids in its identification. In **Figure 1C**, the first-generation female sample also showed a clearly defined excretory pore opening and tail end, further supporting species identification. The vulva of the hermaphrodite, depicted in **Figure 1D**, was observed to be slightly protruding, indicating its reproductive stage, which is typical for *H. indica*. The anterior end of the hermaphrodite, as shown in **Figure 1E**, displayed specific morphological characteristics that are essential to distinguish this species from others. These observations collectively confirm the identity and morphological traits of *H. indica*.

The morphometric analysis of *H. indica VLEPN01*, compared to the Indian isolate (CH7) and the type specimen, reveals several notable differences. For hermaphrodites, *VLEPN01* exhibits a mean body length of 2,800 ± 57 μm, which is smaller than *CH7* (3,476 ± 401 μm) but comparable to the type specimen (2,700 ± 1,000 μm). The mid-body diameter (MBD) of *VLEPN01* (135 ± 5.1 μm) is also smaller than that of *CH7* (245 ± 53 μm), though it aligns closely with the type specimen (132 ± 9 μm). The excretory pore to anterior end (EP) distance in *VLEPN01* (176 ± 5.8 μm) is greater than *CH7* (147 ± 9.0 μm) but similar to the type specimen (173 ± 8 μm). Notably, the percentage of EP relative to the pharynx length (D%) is higher in *VLEPN01* (105 ± 119) compared to *CH7* (88 ± 4.4), with data for the type specimen not available (**Table 1**).

**Table 1:** Morphometrical characterization of the isolated *Heterorhabditis indica* VLEPN01, along with comparisons to the Indian isolate (CH7) and the type specimen.

Character	*H. indica* VLEPN01 (mean ± SD, μm) Present study	Indian isolate (CH7) (mean ± SD, μm) Bhat et al., 2021	Type specimen (original) (mean ± SD, μm) Poinar et al., 1992
Hermaphrodite			
L	2,800 ± 57 (2,400–3,300)	3,476 ± 401 (2,861–4,227)	2,700 ± 1,000 (2,300–3,100)
MBD	135 ± 5.1 (105–148)	245 ± 53 (140–345)	132 ± 9 (107–145)
EP	176 ± 5.8 (165–182)	147 ± 9.0 (128–174)	173 ± 8 (163–187)
NR	118 ± 6.1 (101–125)	131 ± 7.0 (119–146)	115 ± 5 (104–123)
PS	175 ± 5.2 (165–180)	175 ± 6.9 (165–186)	172 ± 6 (163–179)
T	89 ± 4.8 (75–105)	91 ± 12 (79–114)	92 ± 11 (72–110)
D%	105 ± 119 (115–3.9)	88 ± 4.4 (81–100)	–
Female (second generation)			
L	1,500 ± 4.7 (1,300–1,700)	1,434 ± 17 (1,274–1,993)	1,600 ± 12 (1,200–1,800)
MBD	99 ± 4.9 (79–117)	91 ± 17 (70–135)	95 ± 15 (107–145)
EP	129 ± 7.2 (119–141)	115 ± 7.4 (105–129)	127 ± 4 (163–187)
NR	90 ± 3.9 (87–95)	95 ± 6.3 (84–111)	92 ± 4 (104–123)
PS	129 ± 6.5 (118–137)	133 ± 7.7 (124–155)	131 ± 4 (163–179)
T	75 ± 3.8 (62–80)	75 ± 5.9 (64–83)	76 ± 9 (72–110)
D%	89 ± 3.5 (72–93)	87 ± 5.6 (77–99)	–
Male			
L	730 ± 56 (575–780)	755 ± 38 (609–916)	721 ± 64 (573–788)
MBD	41 ± 7.1 (34–48)	37 ± 6.1 (26–50)	42 ± 7 (35–46)
EP	128 ± 4.8 (110–140)	92 ± 6.8 (78–109)	123 ± 7 (109–138)
NR	73 ± 5.6 (74–87)	76 ± 3.6 (62–83)	75 ± 4 (72–85)
PS	106 ± 4.6 (94–110)	101 ± 4.1 (90–116)	101 ± 4 (93–109)
T	30 ± 6.1 (26–35)	26 ± 2.5 (18–33)	28 ± 2 (24–32)
Anal body diameter	27 ± 1.5 (25–31)	–	–
E%	106 ± 4.9 (96–135)	369 ± 34 (295–511)	–
Infective juveniles			
L	558 ± 26 (489–573)	565 ± 28 (516–598)	528 ± 26 (479–573)
MBD	21 ± 1.2 (20–24)	22 ± 4.5 (21–25)	20 ± 6 (19–22)
EP	75 ± 1.9 (73–87)	105 ± 6.0 (98–123)	98 ± 7 (88–107)
NR	91 ± 5.3 (88–109)	91 ± 4.6 (82–101)	82 ± 4 (72–85)
PS	121 ± 3.9 (110–125)	117 ± 5.0 (102–129)	117 ± 3 (109–123)
T	92 ± 4.8 (96–111)	100 ± 7.2 (80–112)	101 ± 6 (93–109)
A	25 ± 1.6 (26–28)	25 ± 4.1 (24–27)	26 ± 4 (25–27)
B	4.5 ± 0.8 (4.7–4.9)	4.8 ± 0.2 (4.5–5.4)	4.5 ± 0.3 (4.3–4.8)
C	4.3 ± 1.1 (4.6–4.9)	5.7 ± 0.5 (4.9–7.5)	5.3 ± 0.5 (4.5–5.6)

L, total body length; MBD, mid-body diameter; EP, excretory pore to anterior end; NR, nerve ring to the anterior end; PS, pharynx length; T, tail length; SL, spicule length; GL, gubernaculum length; A, L/MBD; B, L/PS; C, L/T; D%, EP/PS × 100; E%, EP/T × 100.

In the second generation of female samples, *VLEPN01* has a body length of 1,500 ± 4.7 μm, which is slightly larger than *CH7* (1,434 ± 17 μm) but smaller than the type specimen (1,600 ± 12 μm). The mid-body diameter (99 ± 4.9 μm) and other measurements such as EP (129 ± 7.2 μm) and pharynx length (129 ± 6.5 μm) are comparable to those of *CH7* and the type specimen.

For male samples, *VLEPN01* shows a body length of 730 ± 56 μm, which is slightly smaller than *CH7* (755 ± 38 μm) but similar to the type specimen (721 ± 64 μm). The mid-body diameter (41 ± 7.1 μm) and excretory pore to the anterior end (128 ± 4.8 μm) are similar to the type specimen but differ from *CH7*. The infective juveniles of *VLEPN01* have a body length of 558 ± 26 μm, comparable to *CH7* (565 ± 28 μm) and slightly larger than the type specimen (528 ± 26 μm). The percentage of EP relative to the tail length (E%) in *VLEPN01* is notably lower (106 ± 4.9) compared to *CH7* (369 ± 34), indicating possible differences in developmental or environmental adaptation. The morphological data of *VLEPN01* across different life stages closely align with those of the type specimen and previously identified Indian isolates of *H. indica*, confirming the successful isolation and identification of this strain.

### 3.4 Principal component analysis of morphometric data for *Heterorhabditis indica VLEPN01* across developmental stages

**Figure 2:**
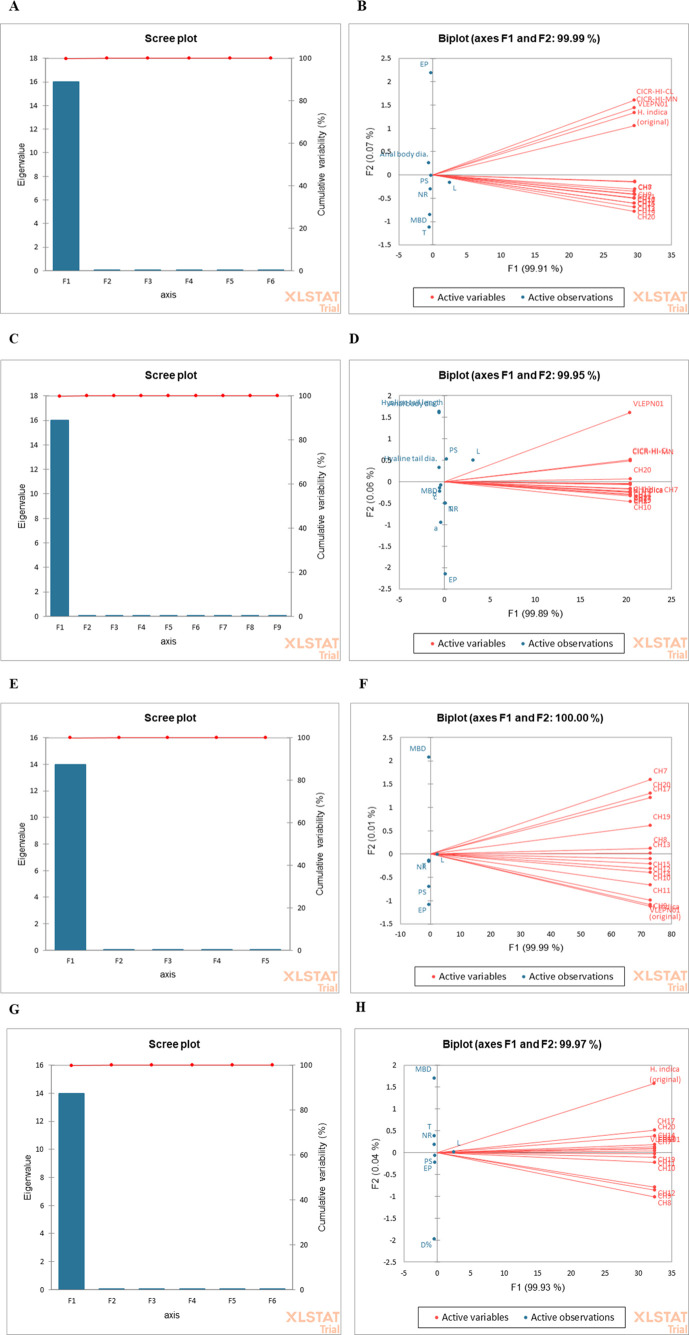
Principal component analysis (PCA) of morphometric data for *Heterorhabditis indica* isolates across developmental stages. **(A, B)** PCA biplot for male stage. The plot demonstrates distinct clustering patterns, indicating intraspecific variability and highlighting differences in body length, spicule length, tail length, and excretory pore distance among isolates. **(C, D)** PCA biplot for infective juveniles (IJs). The plot highlights significant differences in body length, esophagus length, tail length, and excretory pore distance among isolates. **(E, F)** PCA biplot for hermaphrodites. The plot reveals a clear separation of isolates based on body size, esophageal structure, anal body width, and EP distance. **(G, H)** PCA biplot for females. The biplot highlights the unique morphometric identity of the VLEPN01 isolate compared to other female isolates.

The PCA of morphometric data for various stages of *H. indica VLEPN01* revealed significant variability in comparison to other isolates from the Northwestern Himalayas region (**Figure 2**). For the male stage, the PCA identified two principal components (PC1 and PC2) that together explained 70% of the variance (**Figures 2A, B**). PC1 was primarily influenced by body length and spicule length, while PC2 was dominated by tail length and the distance to the excretory pore (EP). The analysis highlighted substantial intraspecific variability, indicating potential adaptations and differences in reproductive strategies and ecological niches. For infective juveniles (IJs), the PCA showed that PC1 accounted for 48.7% of the variance (**Figures 2C, D**), primarily influenced by body length and esophagus length, while PC2 explained 21.3% of the variance, driven by tail length and the distance to the EP. This differentiation underscored the unique morphometric characteristics of *H. indica* isolates compared to others. In the hermaphrodite stage, the PCA revealed that PC1 (45.7% variance) was influenced by body length, esophageal length, and tail length, while PC2 (19.6% variance) was associated with anal body width and EP distance (**Figures 2E, F**). The distinct clustering of *H. indica VLEPN01* confirmed its unique morphometric identity, emphasizing traits like body size and esophageal structure. For the female stage, the PCA demonstrated that PC1 (55% variance) was driven by body length and vulva-to-anus distance, while PC2 (30% variance) was associated with body width and tail length (**Figures 2G, H**). The analysis indicated that the *VLEPN01* isolate had distinct characteristics with longer body size and reproductive distance compared to other isolates, suggesting adaptations or evolutionary changes. The PCA effectively highlighted significant morphometric differences among *H. indica* isolates, providing insights into their ecological and biological variations.

### 3.5 Molecular characterization and phylogeny estimation

**Figure 3:**
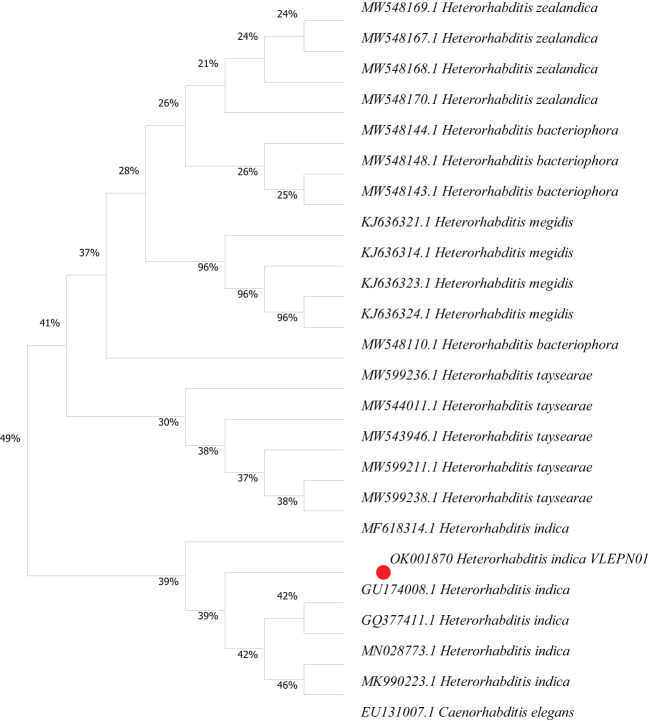
Phylogenetic tree analysis of ITS sequences from *Heterorhabditis indica*. The maximum likelihood phylogenetic tree was constructed based on the ITS sequences (786 bp) of *Heterorhabditis indica*, including the native entomopathogenic nematode isolate and reference sequences. Bootstrap values are shown at the nodes to indicate the robustness of the inferred relationships. The tree was rooted using a *Caenorhabditis elegans* as outgroup. The branches are labeled with species names and accession numbers. The native EPN isolate (OK001870) clusters closely with *Heterorhabditis indica* isolates from Mizoram, Northeastern India (MF618314.1).

The molecular characterization of the isolated strain revealed that its DNA sequence, spanning 786 base pairs, shared an impressive 99.87% similarity with a known strain of *H. indica* from Mizoram, the Northeastern Himalayas region of India (MF618314). This identification was based on the 18S rRNA SSU (small subunit) ITS (internal transcribed spacer) marker, which was used in the PCR analysis. The findings suggest the close genetic relationship between the native EPN isolate and the *H. indica VLEPN01* from the region, suggesting that they likely belong to the same species and are closely related variants. Such genetic insights are crucial for understanding the diversity and evolutionary relationships within these beneficial nematodes, essential for potential applications in biological control and agriculture. The ITS sequence (786 bp) obtained from the native EPN isolate was aligned with homologous sequences from related *Heterorhabditis* species, including *H. indica* (MF618314) from Mizoram, northeastern India, and other closely related taxa. The gene sequence has been successfully submitted at NCBI with accession ID OK001870. The phylogenetic-tree-based evolutionary relationships (**Figure 3**) reveal that the native EPN isolate (OK001870) clustered distinctly within the clade containing *H. indica* isolates. The phylogenetic analysis supports the close genetic relationship between the native EPN isolate and *H. indica* from Mizoram, indicating that they likely belong to the same species and closely related variants within the genus *Heterorhabditis* (**Figure 3**). The bootstrap analysis provided strong support for the inferred relationships within the phylogenetic tree.

### 3.6 Biocontrol potential of *Heterorhabditis indica* against insect pests

**Figure 4:**
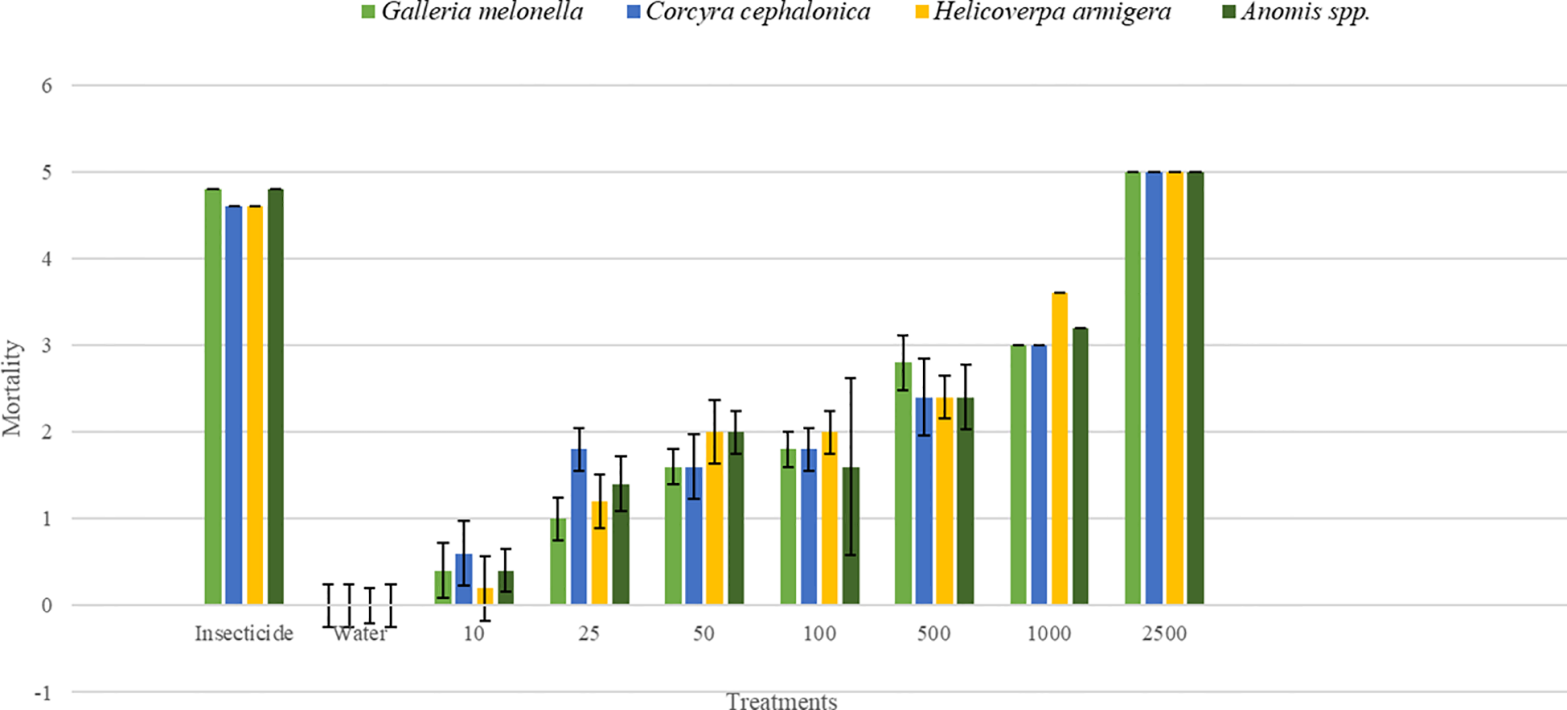
Larvicidal activity of *Heterorhabditis indica* against various insect pests prevalent in the Northwestern Himalayas region. The figure illustrates the larvicidal activity of *H. indica* against four insect species: *Galleria melonella*, *Corcyra cephalonica*, *Helicoverpa armigera*, and *Anomis* spp. Different concentrations of IJs per milliliter (mL) were tested, ranging from 0 to 2,500 IJs/mL. Each mortality value has been scaled, where 5 = 100%, 4 = 80%, 3 = 60%, 2 = 40%, 1 = 20%, and 0 = 0.

The insect’s larvicidal efficacy of *H. indica* was evaluated against different insect species prevalent in the NWH region. The results were scaled with “5” representing 100% mortality and “0” indicating no mortality. For *G. mellonella*, mortality started at 20% with 10 IJs/mL and increased with concentration, reaching 100% at 2,500 IJs/mL and above. *C. cephalonica* showed a similar pattern, with 20% mortality at 10 IJs/mL and 100% mortality from 2,500 IJs/mL onward. *H. armigera* displayed mortality beginning at 20% with 10 IJs/mL, reaching 100% at 5,000 IJs/mL and above, with 80% mortality observed at 1,000 IJs/mL. *Anomis involuta* also followed this trend, with 20% mortality at 10 IJs/mL and 100% mortality at 2,500 IJs/mL and higher concentrations (**Figure 4**).

**Figure 5:**
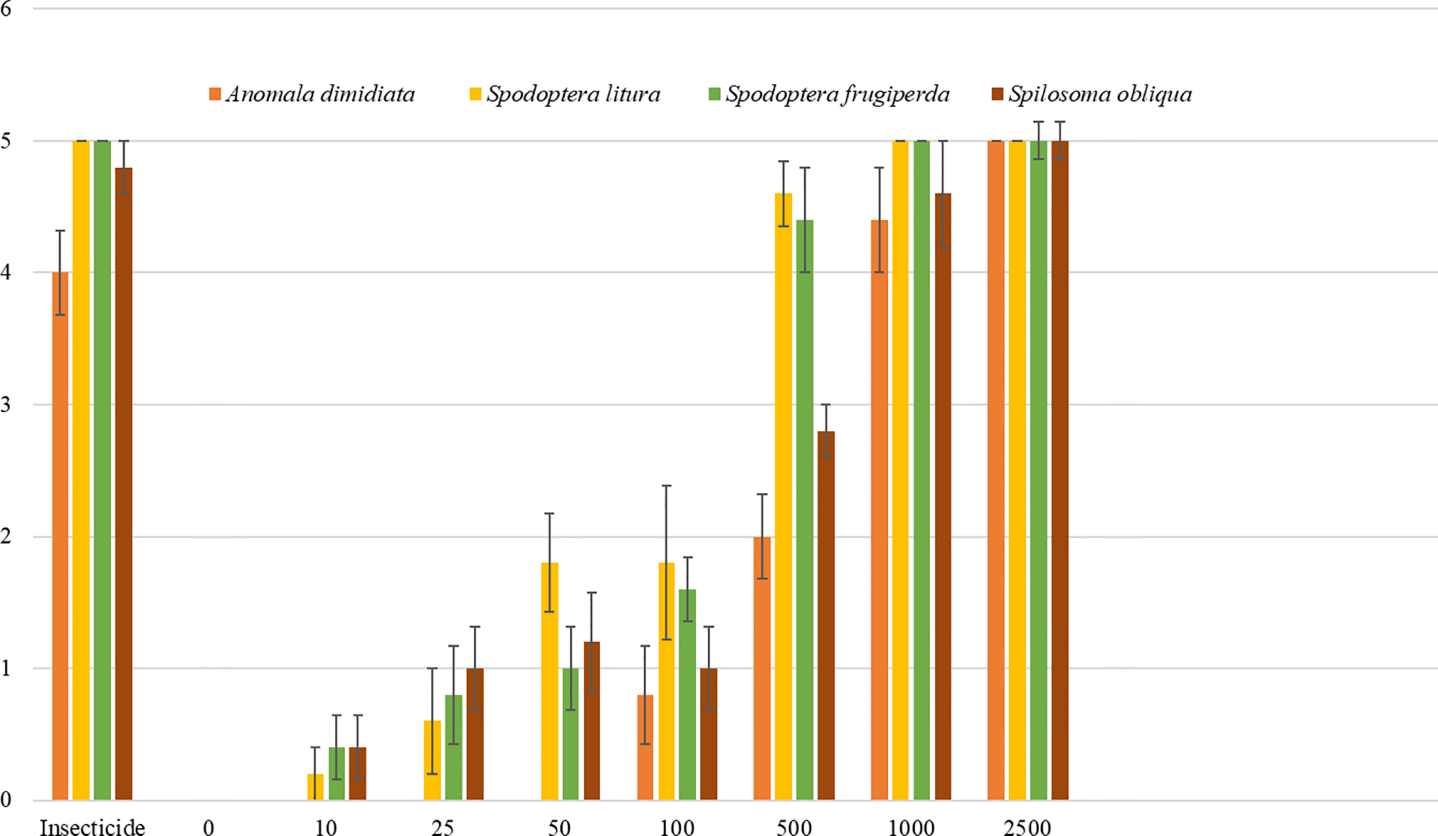
Larvicidal activity of *Heterorhabditis indica* against various insect pests prevalent in the Northwestern Himalayas region. The figure illustrates the larvicidal activity of *H. indica* against four insect species: *Anomala dimidiata*, *Spodoptera litura*, *Spodoptera frugiperda*, and *Spilosoma obliqua*. Different concentrations of infective juveniles (IJs) per milliliter (mL) were tested, ranging from 0 to 2,500 IJs/mL. Each mortality value has been scaled, where 5 = 100%, 4 = 80%, 3 = 60%, 2 = 40%, 1 = 20%, and 0 = 0.

For *A. dimidiata*, mortality was not observed at lower concentrations (10 to 50 IJs/mL) but began at 40% with 500 IJs/mL, reaching 100% at concentrations of 2,500 IJs/mL and above. *S. litura* showed a gradual increase in mortality starting at 20% with 10 IJs/mL and achieving full mortality at 1,000 IJs/mL. *Spodoptera frugiperda* exhibited a similar trend, with mortality starting at 20% with 10 IJs/mL and reaching 100% at 1,000 IJs/mL. *S. obliqua* had a mortality of 20% at 10 IJs/mL, increasing steadily, with full mortality achieved at 2,500 IJs/mL (**Figure 5**).

## 4 Discussion

The soil sample collection and baiting approach in this study provided critical insights into the presence and distribution of EPNs in the NWH region. Out of the 40 soil samples, only one sample tested positive for EPN. This finding, while indicating a relatively low prevalence of EPN in the sampled areas, suggests the importance of targeted sampling and the potential for EPNs to inhabit specific ecological niches within agro-ecosystems (Mukherjee and Ray, 2024). The climatic conditions during the sampling period reflect a warm and humid environment that is generally favorable for nematode activity (Bakonyi and Nagy, 2000; Papatheodorou et al., 2004; Robinson, 2004). These conditions are conducive to the survival and efficacy of EPNs, which require adequate moisture for the mobility and infection of host insects (Abate et al., 2019; Ramakrishnan et al., 2022). The EPN-positive sample was located at geo-coordinates 79°37′52.071″ E, 29°38′0.783″ N, at an elevation of 1,220 m above sea level (mASL), within an agro-ecosystem associated with fodder crops. The presence of white grub insects indicates a suitable habitat for EPNs, as these nematodes are known to target soil-dwelling insect pests. The physico-chemical properties of the EPN-positive soil sample provide valuable information about the specific soil conditions that favor EPN survival and activity (**Supplementary Table S3**). The sandy loam texture suggests good drainage and aeration, which are essential for nematode movement and infection processes (El-Borai et al., 2012). The slightly acidic pH of 6.59 falls within the optimal range for many EPN species (Kung et al., 1990; Gaugler et al., 1997), supporting their viability and efficacy. The low electrical conductivity indicates minimal salinity stress, which can be beneficial for nematode activity. High salinity levels can adversely affect nematode survival and infectivity, making low EC soils more favorable environments for EPNs (Matuska-Łyżwa et al., 2024). The organic carbon content indicates a moderate level of organic matter, which can enhance microbial activity and provide a favorable environment for EPNs by supporting a diverse microbial community that may include symbiotic bacteria essential for EPN survival and reproduction. The successful recovery of EPNs from this soil sample highlights the potential presence and distribution of EPNs in the agro-ecosystems of the study area. We acknowledge that our study was limited to a narrow sampling period (June to October), which may not capture seasonal variations in *H. indica* occurrence. Future studies should incorporate multi-seasonal sampling to assess population dynamics across different climatic conditions.

The detailed morphological and morphometrical characterization of the isolated EPN *H. indica VLEPN01* from the NWH region revealed significant insights into the developmental stages of native EPNs. The measurements obtained for the various developmental stages—hermaphrodites (first generation), female (second generation), male, and IJs—not only provided a comprehensive profile of the nematode but also confirmed the identity of the species as *H. indica*. The observed body length and greatest diameter of the hermaphrodites were consistent with previously reported dimensions (Spiridonov and Subbotin, 2016; Bhat et al., 2021). Morphometrical parameters, such as the measurements from the anterior end to the excretory pore, nerve ring, and the pharynx length, were crucial in distinguishing the developmental stages. The differences in morphometry are indicative of the distinct morphological adaptations at various life stages, essential for their role in parasitism and reproduction (Sudhaus, 2008; Trejo-Meléndez et al., 2024; Mallikarjun et al., 2024). The tail length and diameter at the anal body also provided key differentiators among the stages, with hermaphrodites and IJs exhibiting longer tails compared to female and male samples. This trait is particularly important for the IJs, as the tail morphology plays a vital role in their infective capabilities (Griffin, 2012; Vlaar et al., 2021). The IJs were further analyzed for specific parameters such as the hyaline tail length and diameter. These metrics are essential for understanding the infective potential and survival strategies of the IJs in the soil environment (Khumalo et al., 2021). The distinct morphological and morphometrical characteristics observed in this study are in strong agreement with the descriptions of *H. indica* provided by Poinar et al. (1992); Spiridonov and Subbotin (2016), and Bhat et al. (2021).

The PCA of morphometric data across various developmental stages of *H. indica VLEPN01* from the Northwestern Himalayas region revealed substantial variability and distinct patterns among Indian isolates. For male samples, the PCA identified two principal components that together explained 70% of the variance, with PC1 influenced by body length and spicule length and PC2 by tail length and distance to the excretory pore (EP). This suggests significant intraspecific variability, likely reflecting adaptations to diverse ecological niches and differences in reproductive strategies. In infective juveniles (IJs), PC1 and PC2 together accounted for 70% of the variance, highlighting that body length and esophagus length are key differentiators, while tail length and EP distance also contribute to morphometric divergence. This suggests the unique characteristics of *H. indica* isolates compared to other nematodes, emphasizing specific traits such as body and esophagus length. For the hermaphrodite stage, the PCA showed that PC1 was driven by body length, esophageal length, and tail length, whereas PC2 was influenced by anal body width and EP distance. This analysis confirmed the distinct morphometric identity of the *H. indica* isolate, particularly in terms of body size and esophageal structure. In the female stage, the PCA revealed that body length and vulva-to-anus distance were major factors in PC1, while PC2 was associated with body width and tail length. The VLEPN01 isolate’s distinct profile, with a longer body size and reproductive distance, suggests potential adaptations or evolutionary changes. Overall, PCA effectively highlighted the morphometric diversity among *H. indica* isolates, offering insights into their ecological and biological variations and underscoring the importance of specific morphometric traits in understanding their adaptability and reproductive strategies.

The molecular characterization and phylogeny estimation of the isolated EPN, *H*. *indica VLEPN01*, have provided substantial insights into its genetic identity and evolutionary relationships. The DNA sequence analysis, based on the 18S SSU-ITS marker, revealed a remarkable 99.87% similarity with a known strain of *H. indica* from Mizoram, northeastern Himalayas (MF618314). This high degree of similarity confirms the close genetic relationship between the native EPN strain *VLEPN01* and the Mizoram *H. indica* strain, suggesting that they belong to the same species and represent closely related variants. The ITS sequence alignment with homologous sequences from related *Heterorhabditis* species further corroborates this finding. The successful submission of the gene sequence to NCBI (accession number: ID OK001870) ensures the availability of this genetic data for future reference and comparative studies.

The phylogenetic tree constructed based on these sequences clearly places the native EPN strain *VLEPN01* within the clade containing *H. indica* isolates, providing robust evidence for their close genetic affiliation. The close genetic relationship between the isolate *H*. *indica VLEPN01* and the *H. indica* strain from Mizoram suggests the potential for a broader geographical distribution of this species within the Himalayan region. It also highlights the genetic stability of *H. indica* across different ecological zones, which is promising for its use in biocontrol strategies. The genetic homogeneity suggests that the biopesticidal properties observed in one region can likely be replicated in another, enhancing the practical utility of *H. indica* in pest management (Stiling and Cornelissen, 2005). Combining molecular and morphological data strengthens the taxonomic resolution and ensures accurate species identification, which is vital for effective biological control applications (De Ley et al., 2005; Ahmed et al., 2022).

The bioassay results suggest the significant larvicidal potential of *H. indica* against a variety of insect pests common in the NWH region. The dose-dependent response observed across different species including *G. mellonella*, *C. cephalonica*, *H. armigera*, and *Anomis involuta* demonstrated complete mortality at higher concentrations, showcasing the nematode’s larvicidal activity against these pests. This aligns with previous studies confirming the efficacy of EPNs in controlling these species (Rani et al., 2023; Gokte-Narkhedkar et al., 2005; Lavhe et al., 2023; Gokte-Narkhedkar et al., 2019).

Conversely, the white grub (*A. dimidiata*) required higher concentrations for effective control, with full mortality achieved only at 2,500 IJs/mL. This suggests that *A. dimidiata* might be less susceptible to lower doses, necessitating higher application rates for adequate pest management. Similarly, *S. litura* and *S. frugiperda* also showed high susceptibility but required concentrations of 1,000 IJs/mL for complete mortality, which is slightly lower compared to other pests. *Spodoptera obliqua* exhibited a more gradual increase in mortality, achieving full efficacy only at 2,500 IJs/mL. This variability in susceptibility among different insect species indicates that pest-specific application rates are crucial to optimize the effectiveness of *H. indica VLEPN01*. These findings are consistent with the literature supporting the virulence of EPNs against various insect pests (Chandel et al., 2019; Lalitha et al., 2018; Garcia et al., 2008; Shinde et al., 2023; Pal et al., 2012; Gowda et al., 2020; Dass et al., 2024).

The findings confirm *H. indica VLEPN01*’s significant larvicidal potential of major insect pests prevalent in the NWH. The consistent pattern of increased mortality with higher concentrations reinforces the viability of *H. indica* for integrated pest management strategies, particularly in managing diverse pest species. The EPNs are adapted to the local climate, remain persistent in the soil, and are more suitable as successful biocontrol agents than commercially available strains, as they exhibit higher survival rates, better host-finding abilities, and enhanced efficacy in controlling target pests under specific environmental conditions (Shields et al., 2009; Askary et al., 2017). While the experiments were conducted in a controlled condition, they provide a crucial foundation to understand the virulence and efficacy of this native EPN strain. The observed pattern of increased pest mortality with higher concentrations highlights its biocontrol potential, warranting further field validation. Future research should focus on field evaluations and comparison with commercial strain to confirm the effectiveness of *H. indica VLEPN01* under natural conditions. Additionally, integrating this strain with traditional pest management practices used by local farmers can enhance its impact while ensuring ecological sustainability. Developing cost-effective mass production techniques tailored to the Himalayan region will also be essential for large-scale implementation.

## 5 Conclusion

Entomopathogenic nematodes belong to the families Steinernematidae and Heterorhabditidae and are potential biocontrol agents for the management of a wide range of insect pests. Our current study highlights the isolation, characterization, and virulence of *H. indica VLEPN01* from the NWH region. The findings reveal that *H. indica* exhibits potent larvicidal activity, with dose-dependent mortality across evaluated insect-specific species, including *G. mellonella*, *C. cephalonica*, *H. armigera*, *Spilosoma obliqua*, *Spodoptera litura*, *Anomala dimidiata*, *Spodoptera frugiperda*, and *Anomis involuta*. The effectiveness of *H. indica VLEPN01* is notably higher at the increasing concentration of IJs suspension, confirming its potential as a biological control agent. However, the variability in mortality rates among different pest species, such as the higher concentrations needed for *A. dimidiata*, suggests the importance of tailoring application rates for optimal pest management.

The study also emphasizes the critical role of soil and climatic conditions in EPN distribution and efficacy. The successful isolation of *H. indica VLEPN01* from a specific soil sample, combined with detailed morphological, morphometric, and molecular characterization, provides a comprehensive understanding of its identity and potential. The close genetic relationship with other *H. indica* strains from different regions further supports the species’ adaptability and highlights its potential for broader application across the Himalayan region. Future research should focus on optimizing the use of *H. indica VLEPN01* by studying its adaptation to local climatic and soil conditions, integrating it with traditional pest management practices, and developing sustainable mass production methods suited to the Himalayan environment. To establish the field efficacy and adaptability of the newly isolated *H. indica* strain, multilocational field trials across diverse agro-ecological zones of the Mid-Indian Himalayan region are warranted. These steps are essential to enhance and investigate the practical utility of *H. indica VLEPN01* in integrated pest management strategies and to ensure its effectiveness in diverse agroecosystems.

## Data Availability

The datasets presented in this study can be found in online repositories. The names of the repository/repositories and accession number(s) can be found below: https://www.ncbi.nlm.nih.gov/genbank/, OK001870.
