# Supplementary material for: Isolation, morphological, and molecular characterization of a native Heterorhabditis indica strain from the Mid-Indian Himalayas with insights into biocontrol potential
Source: Front Plant Sci. 2025 Jun 19;16:1576159. doi: 10.3389/fpls.2025.1576159 (PMC12222126; doi:10.3389/fpls.2025.1576159)
Supplement: Supplementary file 1 [file Table1.docx]

**Supplementary table 1.** Geographical locations showing soil sample collection site

| **Locality** | **District** | **Longitude** | **Latitude** | **Altitude (mAMSL)** |
| --- | --- | --- | --- | --- |
| Peora | Almora | 79⁰37’27.676” E | 29⁰32’23.755” N | 1304 |
| Peora | Almora | 79⁰37’39.648” E | 29⁰31’5.889” N | 1424 |
| Talla | Almora | 79⁰37’44.961” E | 29⁰30’59.389” N | 1433 |
| Peora | Almora | 79⁰37’47.740” E | 29⁰31’4.720” N | 1432 |
| Deoli | Almora | 79⁰37’49.983” E | 29⁰31’11.799” N | 1443 |
| Saitoli | Almora | 79⁰38’3.591” E | 29⁰31’8.673” N | 1584 |
| North Gola range | Almora | 79⁰37’45.590” E | 29⁰30’42.118” N | 1637 |
| Saitoli | Almora | 79⁰37’31.928” E | 29⁰30’40.475” N | 1656 |
| Kabhra | Almora | 79⁰37’25.544” E | 29⁰30’43.494” N | 1668 |
| Kabhra | Almora | 79⁰37’20.415” E | 29⁰30’43.831” N | 1680 |
| North Gola range | Almora | 79⁰37’12.902” E | 29⁰30’27.468” N | 1679 |
| North Gola range | Almora | 79⁰37’30.576” E | 29⁰29’37.543” N | 1966 |
| Chausali | Almora | 79⁰37’8.341” E | 29⁰30’17.957” N | 1701 |
| Darima | Nanital | 79⁰37’39.859” E | 29⁰29’14.461” N | 1898 |
| Darima | Nanital | 79⁰38’19.703” E | 29⁰28’10.783” N | 1785 |
| Darima | Nanital | 79⁰38’2.651” E | 29⁰28’2.890” N | 1762 |
| Darima | Nanital | 79⁰38’4.737” E | 29⁰28’1.411” N | 1774 |
| Darima | Nanital | 79⁰38’4.358” E | 29⁰27’56.000” N | 1773 |
| Darima | Nanital | 79⁰38’9.829” E | 29⁰27’52.620” N | 1769 |
| Darima | Nanital | 79⁰38’2.321” E | 29⁰27’49.583” N | 1781 |
| Darima | Nanital | 79⁰38’2.121” E | 29⁰27’47.941” N | 1782 |
| Darima | Nanital | 79⁰38’2.705” E | 29⁰27’45.566” N | 1780 |
| Darima | Nanital | 79⁰38’2.573” E | 29⁰27’34.769” N | 1765 |
| Gargaon | Nanital | 79⁰37’55.164” E | 29⁰28’6.872” N | 1784 |
| Gargaon | Nanital | 79⁰38’10.979” E | 29⁰27’22.077” N | 1779 |
| Darima | Nanital | 79⁰38’7.901” E | 29⁰27’33.849” N | 1759 |
| South gola range | Nanital | 79⁰37’50.647” E | 29⁰27’53.016” N | 1793 |
| South gola range | Nanital | 79⁰38’19.703” E | 29⁰28’10.783” N | 1802 |
| South gola range | Nanital | 79⁰38’3.884” E | 29⁰27’4.570” N | 1816 |
| South gola range | Nanital | 79⁰38’8.904” E | 29⁰27’2.571” N | 1826 |
| South gola range | Nanital | 79⁰38’12.927” E | 29⁰27’5.372” N | 1867 |
| Gangachor | Nanital | 79⁰38’38.268” E | 29⁰26’2.905” N | 2082 |
| Mukteshwar | Nanital | 79⁰38’57.658” E | 29⁰26’35.691” N | 2157 |
| Mukteshwar | Nanital | 79⁰39’4.192” E | 29⁰26’40.410” N | 2158 |
| Mukteshwar | Nanital | 79⁰39’9.397” E | 29⁰26’45.880” N | 2171 |
| Sargao khet | Nanital | 79⁰39’19.347” E | 29⁰27’4.852” N | 2205 |
| Sargakhat | Nanital | 79⁰39’17.479” E | 29⁰27’20.147” N | 2240 |
| Jaspur | Nanital | 79⁰38’45.103” E | 29⁰28’29.306” N | 2295 |
| Hawalbagh | Almora | 79⁰37’52.071” E | 29⁰38’0.783” N | 1220 |
| Hawalbagh | Almora | 79⁰37’51.679” E | 29⁰37’58.883” N | 1220 |

**Supplementary table 2:** Physico-chemical properties of EPN positive sample.

| **Parameter** | **Unit** | **Control** | **Method** |
| --- | --- | --- | --- |
| pH | - | 6.59 | 1:2 soil-water suspension method |
| EC(1:2 soil-water suspension) | dSm^-1^ | 0.105 | 1:2 soil-water suspension method |
| Organic carbon | g kg^-1^ | 10.6 | Modified Walkley & Black method |
| Mineralizable-N | kg ha^-1^ | 467.6 | Subbiah & Asija method |
| Available P | kg ha^-1^ | 49.32 | Olsen method |
| Available K | kg ha^-1^ | 128.2 | Ammonium Acetate method |
| Available - Zn | mg kg^-1^ | 2.77 | DTPA-CaCl_2_ –TEA extraction method |
| Available -Mn | mg kg^-1^ | 5.4 |  |
| Available -Cu | mg kg^-1^ | 1.78 |  |
| Available -Fe | mg kg^-1^ | 24.7 |  |
| Soil Texture | - | Sandy loam | Mason jar soil test method |

**Table 1.** Artificial diet of *Galleria melonella* for box^-1^

| **Ingredient** | **Qty (gram)** |
| --- | --- |
| Maize flour | 100 |
| Wheat | 25 |
| Milk powder | 50 |
| Wheat bran | 50 |
| Wax | 25 |
| Yeast powder | 25 |
| Honey | 56.25 ml |
| Glycerol | 56.25 ml |
